# The Effect of Loving-Kindness Meditation on Flight Attendants’ Spirituality, Mindfulness and Subjective Well-Being

**DOI:** 10.3390/healthcare8020174

**Published:** 2020-06-16

**Authors:** Chao Liu, Hao Chen, Chia-Yi Liu, Rung-Tai Lin, Wen-Ko Chiou

**Affiliations:** 1 College of Aviation, Hua Qiao University, Xiamen 361021, China; victory666666@126.com; 2 Graduate Institute of Business and Management, Chang Gung University, Taoyuan 33302, Taiwan; haochen19606@foxmail.com; 3 Department of Psychiatry, Chang Gung Memorial Hospital, Taipei 10507, Taiwan; liucy752@cgmh.org.tw; 4 Graduate School of Creative Industry Design, National Taiwan University of Arts, New Taipei 22058, Taiwan; rtlin@mail.ntua.edu.tw; 5 Department of Industrial Design, Chang Gung University, Taoyuan 33302, Taiwan

**Keywords:** loving-kindness meditation, mindfulness, spirituality, subjective well-being

## Abstract

*Background*: This study investigated: (1) the effects of the loving-kindness meditation (LKM) on mindfulness, subjective well-being (SWB), and spirituality and (2) the relationships between mindfulness, spirituality, and SWB. *Methods*: 98 flight attendants from Xiamen Airlines in China were recruited and randomly assigned to the LKM training group (*n* = 49) or the waiting control group (*n* = 49). The LKM training group underwent an 8-week LKM training intervention, and the control group did not undergo intervention. The three main variables (SWB, mindfulness, and spirituality) were measured both before (pre-test) and after (post-test) the LKM training intervention. *Results*: In the experimental group, SWB and spirituality increased significantly. In the control group, no significant differences were observed for the three variables between the pre-test and post-test. *Conclusions*: Our results indicated that LKM may help to improve SWB and spirituality. However, the mechanisms which underlie the effects of the LKM on mindfulness, spirituality, SWB, and other psychological constructs require further elucidation.

## 1. Introduction

According to the World Health Organization definition of “health”, it not only includes physical health, but also mental health. As an important part of mental health, subjective well-being (SWB) has been paid more and more attention by public health researchers [1,2]. Compared with other occupations, flight attendants have obvious particularities: (1) Working environment. The working scope of flight attendants is limited to the cabin of the aircraft, with narrow space and poor air quality. There are many adverse factors in the work environment, including the high-altitude environment, radiation, aircraft noise, constant turbulence, etc., which may cause the flight attendants physical discomfort, or even psychological irritability [3]. (2) The working hours are not fixed. The working hours of flight attendants change frequently and are usually determined according to the flight schedule, especially for flight attendants on international routes [4]. (3) Negative interactions. Regardless of the interaction with passengers or the particularity of flight attendant occupation, negative emotions such as anxiety and depression are often generated, which lead to fatigue and stress of flight attendants, forming risk factors for their happiness [5]. On the one hand, flight attendants shape the image of the airline, on the other hand, they are also related to the aviation safety. The mental health of flight attendants is very important. In addition to alleviating flight attendants’ anxiety, depression and other negative emotions, it is more important to study whether it can bring SWB to flight attendants from the perspective of positive psychological improvement and understand the reasons for SWB.

The job of flight attendants can be viewed from the perspective of “emotional labor” [6]. In this process, the face and body of flight attendants such as smile, beauty, charm and vitality become a commodity form of airlines. Emotional labor requires flight attendants to induce or suppress their emotions in order to maintain an image that both empathizes and pleases customers. Emotional labor is the effort to understand others, to empathize with their situation, to feel what they feel as part of one’s own [7]. First, mindfulness enables flight attendants to use deep acting in their emotional labor. Deep acting and surface acting are two strategies for flight attendants to provide emotional labor. Surface acting is to pretend to show the required emotions without touching the deep feelings. Mindfulness allows flight attendants to adjust their inner positive attitudes and feelings, use deep acting strategies, and be happier at work [6]. Secondly, spirituality can ease the conflicts between flight attendants and passengers in the process of emotional labor. When flight attendants conflict with passengers and are upset by passengers, they can find refuge in the connection of transcendence and treat suffering as an illusion, unreal and short-term disturbance that causes confusion. The flight attendants are encouraged to imagine the troubled passengers as suffering people, who are ignorant people who need help [8]. Finally, SWB can maintain the flight attendant’s happy facial expression in the process of emotional labor. In the airline service industry, the sustained happy facial expression of the flight attendants is a necessary condition for building a friendly passenger relationship. A happy face reflects the service quality of the airline. A deadpan face can mean unfriendly behavior for passengers, and SWB can maintain the happy facial expressions of flight attendants at work [9]. The loving-kindness meditation (LKM) helps to open one’s heart, to experience and perceive the feelings of others and oneself in a loving, kind, peaceful and considerate way, and to generate and cultivate qualities and emotions such as acceptance and care [10]. Through the perception and awareness of negative emotions, LKM has a regulating effect by directly transforming negative emotions into positive ones [11]. Due to the nature of work, flight attendants generally have the need to actively regulate their emotions. In the selection of specific emotional regulation strategies, they tend to avoid using those that consume physical and cognitive resources [8]. LKM converts emotions directly without the need for an intermediate cognitive reevaluation process, and the practice content and method of LKM are easy to understand and operate [12], which are consistent with the characteristics of flight attendants. Therefore, LKM may be an effective way to help flight attendants regulate and improve their emotions. This study uses LKM as an intervention method and selects the three most important variants of positive psychology for flight attendants: mindfulness, spirituality and SWB.

### 1.1. Subjective Well-Being Theory

SWB primarily refers to how an individual perceives their quality of life, on both a cognitive and emotional level. In this sense, an individual’s happiness or not determined by the actual events of their lives; happiness is determined by the individual’s interpretation of these events and the cognitive and emotional responses they trigger [13]. There are two facets to SWB: emotional balance and life satisfaction. Emotional balance refers to the happy experience that occupies the comparative advantage compared with the unpleasant emotional experience, which is an overall and general evaluation of the individual’s life. Emotional balance comprises both positive and negative emotions. However, these two dimensions are usually unrelated; they are relatively independent variables. Life satisfaction comprises all the perceptions an individual has about their life. As a cognitive factor, life satisfaction is independent of positive emotions and negative emotions. It is a more effective indicator of SWB [14]. SWB is positively correlated with employees’ execution and creativity, and positively correlated with employees’ engagement and loyalty. The higher the SWB, the better the employee performance [15]. SWB has a negative influence on depression and anxiety, and the higher the SWB is, the lower the depression and anxiety is [16]. Given the effects of SWB on physical and mental health, it is important to investigate how this phenomenon enhances emotional and cognitive processes, both of which are related to mindfulness and spirituality [17].

The sustainable happiness model proposed by Lyubomirsky et al. [18] is one of the important theoretical bases for SWB promotion. The model contains three factors: genetic set points, living circumstances and intentional activities. A genetic set point is a stable, long-term baseline of personal SWB. Twin studies pointed to the long-term stability of happiness. In the study of twins, the cross-time correlation coefficient of identical twins’ SWB reached 0.40, indicating that the influence of genetic factors on individual SWB is long-term and stable [19]. Everyone’s baseline happiness is relatively stable, and although it may fluctuate in the short term, it will revert to the baseline over time. The living circumstance is an occasional but relatively stable factor in an individual’s life. Related to SWB of the living circumstance factors include individual countries, geography, culture environment, and demographic factors such as age, gender and race, also contain individual personal history (i.e., life events that affect their SWB, such as childhood experiences and life accident, etc.), as well as personal life (such as occupation, income, health, faith, etc.) [20]. Intentional activities are the behavior and actions that people choose to do. They require a certain level of effort—that is, the individual clearly takes some action, rather than allowing it to happen naturally. The key difference between intentional activity and living circumstance is that living circumstance is what happens to people, while the intentional activities is people’s behavior of actively changing the circumstances [18].

For the promotion of SWB, the genetic set point is fixed for a long time and cannot be changed. The living circumstance is influenced by hedonic adaptation. Hedonic adaptation refers to the change of personal status (such as positive events), which can improve people’s SWB in a short period of time, but people will quickly adapt to this change, so that the growth of SWB caused by this change will rapidly decline or even disappear, and SWB will return to the baseline value. Improvements in living conditions, including material, financial, love and other improvements, are easily offset by improvements in people’s SWB as they adapt [18]. Research showed that for most people, the life satisfaction that came with marriage faded over time [21], so while a person may experience a temporary boost in SWB due to events such as a move, a raise in salary, etc., the boost is not permanent. People tend to adapt to constant circumstances, and once the ceiling effect of the positive circumstances appear, it is difficult to further increase SWB. The theoretical starting point of SWB promotion in this study is people’s intentional activities. Both theoretical and empirical studies suggest that people can increase their SWB through simple, positive intentional activities, such as LKM [22]. Through conscious effort, one can alter cognition to counteract the adaptation of these intentional activities to current life events and emotions, thereby avoiding adaptation to SWB.

### 1.2. Loving-Kindness Meditation, Mindfulness, Spirituality and SWB

Loving-kindness meditation (LKM) has been practiced by buddhists for more than 2500 years [23], but its usefulness as a psychological intervention has only recently been explored [24]. Self-compassion consists of three main elements: kindness, a sense of common humanity, and mindfulness. These factors combine and interact to create a state of mind of self-compassion. Self-compassion is relevant when considering one’s own inadequacies, mistakes and failures, as well as in the face of painful life situations which get out of control. Self-compassion therapy is to co-exist with one’s own suffering, and to respond to oneself with kindness and tenderness [25]. LKM is about caring for all living beings besides oneself, the purpose of which is to cultivate feelings of unconditional love, kindness, and acceptance [23]. When practicing LKM, the practitioner directs loving-kindness, in a stepwise fashion, toward themselves, loved ones, acquaintances, strangers, and finally, all sentient beings [26]. LKM is versatile, it can be practiced at any time and in a variety of postures such as lying down, sitting, walking [24]. Compared to traditional meditation methods, LKM is a simpler and easier way, less susceptible to the constraints of space and time, and can be performed at any time and place [24]. Therefore, LKM is more suitable for the nature of flight attendants and will not affect or interfere with their work. When they encounter a sudden situation, they can also use LKM to calm their emotions and resolve conflicts in a timely manner. Previous studies on psychotherapy treatment with LKM have proved its therapeutic effect on different groups. First, the study of LKM applied to the general population found that it can reduce the negative emotions of parenting parents [27], reduced the negative emotions of adolescents [28], reduced the depression and anxiety of self-critics [29], and reduced the post-traumatic stress disorder of veterans [30]. Secondly, the application of LKM to people with physical and mental disabilities found that it can reduce the depression of patients with chronic depression [31], reduce the negative emotions of schizophrenics [32], and reduce the depression and anxiety of patients with borderline personality disorder [12]. Driven by positive psychology, researchers began to shift their attention from negative improvements to positive ones. Therefore, this study chooses LKM as the intervention method for flight attendants, and chooses three positive psychological factors (Mindfulness, spirituality and SWB), which are closely related to flight attendants, as the research variables of this study.

Few studies explore the effects of LKM on mindfulness, spirituality, and SWB. The study of Sorensen shows that LKM can increase of college students’s mindfulness [33]. Mindfulness is defined as focusing purposeful, conscious, and non-judgmental attention/awareness to what is happening in the present moment. In other words, it involves being aware of what’s going on in one’s own consciousness and the surrounding environment without judging it, analyzing it, or reacting to it. There are two core points of mindfulness: one is to focus attention on the present moment; The second is not to evaluate all the concepts at present. That is to cultivate a sense of awareness about the present and maintain an open and accepting attitude. Non-judgement refers to not complaining about yourself, the environment, and others, which is necessary to be fully aware of your current mental and physical feelings or experiences [34]. Previous research have shown that mindfulness increases the SWB of professional athletes [35], professional employees [36], and teachers [37]. Although there is much evidence to suggest that mindfulness has a positive correlation with SWB, research that explores the mechanism which underlies this relationship is limited. It is possible that spirituality can help explain the relationship between mindfulness and SWB, and this topic deserves further investigation.

Spirituality pertains to the connections that one pursues and experiences with the essence of life. It consists of three dimensions: connection with oneself, connection with others and nature, and connection with transcendent experiences [38]. Previous studies have explored spiritual prepositional variables and unearthed that mindfulness can increase individual spiritual growth [39]. Mindfulness training can affect and improve health-related quality of life by enhancing spirituality [40], and mindfulness can improve the spirituality of healthy adults [39,41]. Researchers have further investigated specific groups of people to find that mindfulness can enhance the spirituality of adolescents [42], and have a positive correlation with the spirituality of psychotic patients [43], and even help cancer patients improve spirituality and post-traumatic growth [44,45]. There are also literatures that reveal the positive influence of spirituality on SWB. Studies show that spirituality can enhance the SWB of the elderly [46], have a positive correlation with SWB of adolescents [47]. The researchers further investigated the specificity of the workplace group to find that spirituality has a positive correlation with the SWB of high school teachers [48], American service workers [49], and has a positive correlation with the SWB of Indian business managers [50]. Taken together, these previous findings strongly suggest that individuals with a high degree of mindfulness experience higher levels of spirituality and thereby increase their SWB.

### 1.3. Research Gap, Purpose and Hypotheses

With the development of the world economy, aircraft have become the main mode of transportation for people to travel. In addition to the service, the flight attendant’s work also bears the important responsibility of flight safety which is related to everyone. However, few studies have explored the construction of positive psychological factors for flight attendants and the impact of these psychological factors, this study attempts to fill this gap. Therefore, the purpose of this study is to verify whether LKM can enhance the flight attendant’s mindfulness, spirituality, and SWB.

Based on the above literature theories and research findings, this study proposes the following hypotheses:

**Hypothesis 1** **(H1).** *In LKM group, the post-test scores of the three scales (mindfulness, spirituality and SWB) were significantly higher than the the corresponding pre-test scores.*

**Hypothesis 2** **(H2).** *In control group, there was no significant difference between the post-test scores of the three scales and the corresponding pre-test scores.*

**Hypothesis 3** **(H3).** *The post-test scores of the three scales in LKM group were significantly higher than those in the control group.*

## 2. Methods

### 2.1. Participants

The present study involved 98 flight attendants (22 males, who accounted for 22.44% of the participants; M = 29.26, SD = 6.12) from Xiamen Airlines in China. All participants were aged 21–40 years old. Participants were randomly assigned into one of two groups: the LKM training group (49 participants) and the waiting control group (49 participants). The two groups were not significantly different in terms of demographic characteristics (Table 1).

### 2.2. Instruments

Due to the inability to find the published Chinese translation version which already tested for reliability and validity of SMS and SAIL scales. For this, the SMS and SAIL scales was first translated into Chinese and then back-translated into English (to ensure translation accuracy). Using 98 samples from the data of pre-assessment, this study tested the reliability of the translated Chinese version. The Chinese version of both SMS and SAIL scales showed acceptable reliability.

State Mindfulness Scale (SMS) [51]. This scale is used to measure participants’ state mindfulness. The State Mindfulness Scale (SMS) was developed by Tanay and Bernstein [51] and contains two dimensions: body mindfulness, which involves an awareness of how the body feels and an awareness of sensations within the body; and psychological mindfulness, which involves an awareness of thoughts, images, and emotions that appear in consciousness. The body mindfulness dimension includes 15 items, and the psychological mindfulness dimension includes six items, for a total of 21 items. Participants answer questionnaire items of the SMS using a five-point Likert scale, whereby 1 means “not at all”, and 5 means “very well”. In the SMS, each subscale is individually scored, and the scores of the two subscales are summed to derive a total score. Previous studies have shown that the SMS has good validity [52]. The Cronbach’s alpha of each sub dimension: (1) body mindfulness (0.883); (2) psychological mindfulness (0.897). The Cronbach’s alpha for the overall SMS was 0.894.

Spiritual Attitude and Involvement List (SAIL) [38]. This scale is used to measure the spirituality level of participants and contains seven dimensions: meaning, trust, acceptance, caring for others, connection with nature, transcendence, and spiritual activity [38]. The seven dimensions include a total of 26 items. Participants answer questionnaire items of the SAIL scale using a six-point Likert scale, whereby 1 means “not at all”, and 6 means “very well”. Each subscale is individually scored, and the seven subscales are summed to derive a total score. Previous studies have shown that SAIL has good validity [53]. The Cronbach’s alpha of each sub dimension: (1) meaning (0.876); (2) trust (0.883); (3) acceptance (0.912); (4) caring for others (0.949); (5) connection with nature (0.865); (6) transcendence (0.887); (7) spiritual activity (0.903). The Cronbach’s alpha value for the overall SAIL was 0.892.

Satisfaction with Life Scale (SWLS) [54] and Positive and Negative Affect Scales (PANAS) [55]. As noted, SWB is comprised of two parts: emotional balance and life satisfaction [54]. Therefore, the Positive and Negative Affect scale (PANAS), developed by Watson, Clark, and Tellegen [55], is used to measure participants’ positive and negative emotional experiences; and the Satisfaction with Life Scale (SWLS), developed by Diener et al. [54] is used to measure participants’ satisfaction with their lives.

PANAS contains two dimensions: positive emotional experiences and negative emotional experiences. Each dimension has 10 items, for a total of total 20 items. Participants answer using a five-point Likert scale, whereby 1 means “none at all”, and 5 means “all the time”. Each subscale is individually scored, and the two subscales are summed to derive a total score. Previous studies have shown that PANAS has good validity [56]. The SWLS includes seven items, and participants answer using a seven-point Likert scale, whereby 1 means “strongly disagree”, and 7 means “strongly agree”. Previous studies have shown that that the SWLS has good validity [57]. As recommended by Diener et al. [14], we calculated the scores (PA, NA, and SWLS) for each subscale and the acomposite SWB score (SWBC) by adding the positive affect and life satisfaction items and subtracted the negative affect items. The Chinese version of PANAS, adapted by Sheldon et al. [58], has the same two-factor structure. The Chinese version of SWLS was adapted by Wu and Yao [59], presenting the same single-factor structure. Further analysis also showed that SWLS had significant longitudinal invariance in the psychometric characteristics of the Chinese sample [60]. In the current study, the Cronbach’s alpha of each sub dimension: (1) PA (0.878); (2) NA (0.885); (3) SWLS (0.867). The Cronbach’s alpha coefficient for the overall SWB was 0.874.

### 2.3. LKM Intervention

The LKM intervention entailed five weekly 90-min sessions, which is a group LKM training. Each meeting involved three sequential phases: (1) up to 15 min of psychoeducation on topics such as introducing LKM; (2) 30 min of LKM practice; (3) a final discussion phase lasting up to 45 min, in which participants could share their LKM experiences with the other participants and the instructor. For the LKM intervention, participants were instructed to: (1) sit or lay down with closed eyes and pay attention to their breath and body; (2) imagine receiving kindness, love, and compassion from a loving person; and (3) imagine sending those feelings, in a stepwise fashion, to themselves, their family and friends, their community, all people, and finally, all sentient beings [26]. We also incorporated adaptations of LKM into the meditation program used in our study. For example, participants were encouraged to send loving-kindness toward a difficult person. What’s more, participants were instructed to walk outside while sending loving-kindness toward people, animals, and nature in general. Finally, participants were asked to practice these formal meditations between sessions and to apply loving kindness skills in their workplace.

### 2.4. Procedure and Design

We published recruiting advertisements on the Xiamen Airlines’s internal network, Flight attendants who were interested and qualified in participating in our LKM research provided their registration information.

**Figure 1:**
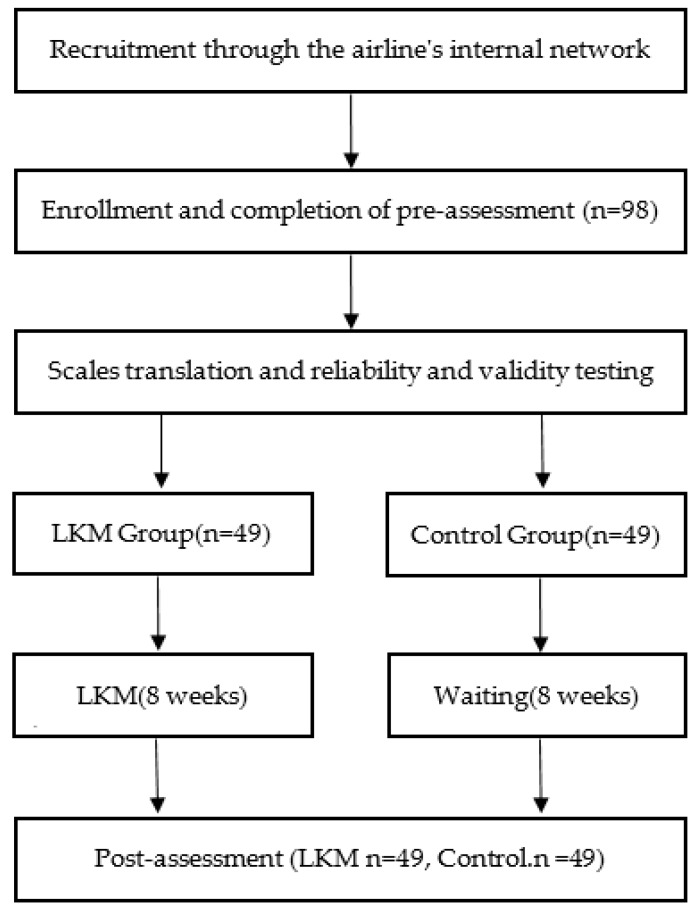
Procedure flow chart.

Inclusion criteria were: (1) adults, ages 18 years or older, (2) able to speak and read Chinese enough to complete the questionnaires. Exclusion criteria were: (1) self-reported diagnosis of depression, anxiety, bipolar disorder, substance abuse or suicide by medical professional, (2) had previously experience about LKM or mindfulness. We then randomly assigned 98 qualified participants were to either the experimental group (i.e., the LKM intervention group, 49 participants) or the control group (i.e., the waiting group, 49 participants). The investigation was conducted by researchers who had 10 years of experience practicing LKM and two years of experience teaching meditation. The sessions (LKM or waiting) were conducted in a quiet, disturbance-free room, and each participant received 50 Chinese Dollars at the start of the investigation to strengthen their motivation. At the beginning of the investigation, each participant was told that they were participating in a personality-related study. Participants were then provided with instructions, and researchers confirmed that participants understood these instructions before continuing. Upon confirming that they understood the instructions, participants provided demographic data and completed the following questionnaires (pre-test): (1) State Mindfulness Scale (SMS); (2) Spiritual Attitude and Involvement List (SAIL); (3) The Positive and Negative Affect Scales (PANAS); and (4) Satisfaction with Life Scale (SWLS). The time required to answer questionnaires was approximately 20 min. Participants then completed a 30 min LKM activity. The duration of the LKM intervention was 8 weeks. At the end of the intervention period, participants completed the same questionnaires again (post-test) and received another 50 Chinese Dollars as compensation. Finally, the true purpose of the study was explained to participants. This research was approved by the Huaqiao University Ethics Committee (IRB No: 201902226B0), and research protocols were carefully reviewed to ensure that they abided by the ethical guidelines of the China Psychological Association (see Figure 1).

Data were analyzed by utilizing three 2 × 2 mixed MANOVAs. The sample size recruited matched or exceeded previous studies. Analyses were conducted by utilizing SPSS version 23 (IBM Inc., Armonk, NY, USA, 2015), a significance threshold was set at *p* < 0.05.

## 3. Results

**Figure 2:**
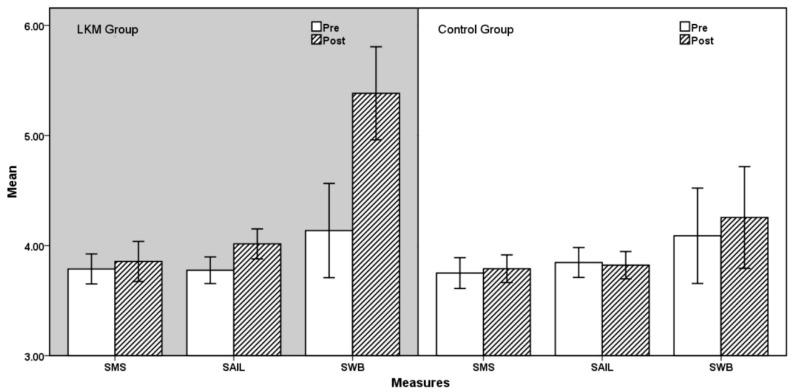
Comparison of three measures between LKM and Control Group. SMS, State Mindfulness Scale; SAIL, Spiritual Attitude and Involvement List; SWB, Subjective Well-Being.

Three 2 (Group Type: LKM, Control) × 2 (Time: Pre, Post) MANOVA with repeated measures on the Time was conducted on the mindfulness (SMS), spirituality (SAIL) and SWB. The *P* values of Box’s Test results of the three groups of data were all greater than 0.05, indicating that the observed covariance matrices of the dependent variables are equal across groups. The details of the results are presented in Table 2 and Figure 2

For mindfulness, there was no significant main effect of Time: F(1,96) = 0.761, *p* = 0.385, and there was no significant main effect of Group Type: F(1,96) = 0.387, *p* = 0.535, and there was also no significant interaction between Time and Group Type, F(1,96) = 0.055, *p* = 0.816 These results showed that there was no significant difference in SMS scores between the pre-test and the post-test, no significant difference between the LKM group and the control group, and no interaction effect in the Time × Group Typ.

For spirituality, there was a significant main effect of Time: F (1,96) = 4.799, *p* = 0.031 η^2^_p_ = 0.048, and there was no significant main effect of Group Type, F (1,96) = 0.638 *p* = 0.426. However, a significant interaction was found between Time and Group Type, F (1,96) = 7.428, *p* = 0.008, η^2^_p_ = 0.070. Although the post-test score of control group was slightly lower than that in the pre-test, the overall result showed a significant main effect of Time due to the improvement of score in the LKM group. Results indicated that LKM produced a significant increase in SAIL score levels.

For SWB, there was a significant main effect of Time: F(1,96) = 24.407, *p* < 0.001 η^2^_p_ = 0.203, and there was a significant main effect of Group Type, F(1,96) = 4.665 *p* = 0.033 η^2^_p_ = 0.046, and there was also a significant interaction was found between Time and Group Type, F(1,96) = 14.285, *p* < 0.001, η^2^_p_ = 0.130. Results indicated that LKM produced a significant increase in SWB score levels.

Our hypothesis stated that LKM should increase mindfulness, spirituality, and SWB. As the results showed that the LKM significantly increased participants’ spirituality and SWB, so Hypotheses 2 was supported, and Hypotheses 1 and 3 were partially supported.

## 4. Discussion

### 4.1. LKM Can Improve Spirituality and SWB

There were significant differences in SWB scores between the post-test and the pre-test, due to the purification of negative emotions, development of positive emotions and improvement of life satisfaction of flight attendants. Firstly, because LKM has the function of purifying negative emotions, the deep meaning of purification is to break through the original psychological dysfunction and achieve spiritual promotion. Flight attendants already have a lot of mental disorders due to their long emotional labor, if they can’t break through barriers, reflected in their consciousness is the everyday experience of many psychological problems, such as depression, anger, hostility and anxiety, most of these psychological problems are maladaptive habitual response patterns fixed by past negative experience. LKM can purify negative emotions in a warm way, and perhaps even replace these negative automatic thoughts with compassion [23]. Secondly, because LKM plays a role in developing positive emotions, according to Fredrickson’s constructive theory of positive emotion extension, positive emotions can expand the attention range of flight attendants and enhance cognitive flexibility [30]. LKM training can effectively cut off the inertial connection of bad emotions, strengthen the positive emotional pattern, especially can dissolve the resentment with great damage to the body and mind, relieve the physical and mental stress, and continuously accumulate positive emotional power. Over time, developing these positive emotions leads to greater happiness and satisfaction. As practice increases, sensitization occurs, meaning that this positive emotion can be more easily approached and triggered at work and in daily life. Finally, because of the role of LKM in promoting life satisfaction, In the process of sending loving-kindness, the participants focused on the sending process, which produced immersion, and even more caused selflessness, not self-centered, generated positive cognition and improved life satisfaction [61]. LKM directly cultivates the four immeasurables: loving-kindness, compassion, appreciative joy, equanimity, then the function of fostering prosocial attitudes goes without saying. In fact, LKM develops the four immeasurables in large part to counter the opposite of the four immeasurables: anger, cruelty, jealousy, and obsession. LKM fosters a positive cognition toward people and oneself, including increased acts of helpfulness, which increase life satisfaction [62].

There were significant differences in spirituality scores between the post-test and the pre-test, due to their improved connection with self, others and transcendence. Firstly, because LKM has the function of improving connection with self, by conveying loving-kindness to himself, the practitioners create a safe space, establish a positive and no defensive attitude to embrace all kinds of experience, and the individual adopts an accepting attitude to all the characteristics of himself and treats the suffering (e.g., hostility, greed, stress and depression) with a kind attitude that is to accept everything in a warm and gentle way [63]. Secondly, because LKM plays an important role in the development of connection with others, LKM tries to encourage practitioners use warm, compassionate feelings towards others, which in turn help practitioners improves the ability of altruism and open attitude. By practicing LKM to cultivate positive attitude towards others, the practitioners are more likely to be altruistic [64]. Finally, because of the role of LKM in improving the connection with transcendence, practitioners gradually minimize themselves from the perspective of self to the perspective of all living beings with practice, thus cultivating non-self and selflessness, and improving the connection with transcendence [65].

We did not observe any significant differences in participants’ mindfulness scores between the pre-test and post-test. Mindfulness involves actively focusing one’s attention and requires clear awareness of internal processes and external environments. The rationale behind mindfulness is that, while the practioner’s attention control intensity typically far exceeds the controlled event, when they are actively practicing the LKM, they are fully immersed in the process of sending positive emotions to themselves and others. In this state of immersion, external awareness is greatly reduced. When the practitioner concentrates on the LKM, perception of the inner perception and the environment are also affected. Previous studies have also reported that immersion and mindfulness are inversely correlated [66], which may explain why we found that the LKM was unable to significantly increase mindfulness. From the perspective of the object, the object of mindfulness meditation is focused, while the object of LKM is rough and open. When practicing loving-kindness, the object of meditation is constantly changing. Thus, the LKM can increase mental disorganization and mental distraction. If one wants to use the LKM to increase mindfulness, the objects of meditation should not be changed quickly, as doing so is detrimental to mindfulness [67].

### 4.2. Research Limitations and Future Studies

As a group engaged in a particular occupation, the flight attendant’s work may cause greater psychological stress. Therefore, the purpose of this study is to investigate whether LKM can increase the mindfulness, SWB and spirituality of flight attendants. The results showed that LKM could improve the spirituality and SWB of flight attendants. Therefore, airlines managers should appropriately promote LKM interventions to enhance flight attendants’ ability to withstand pressure and self-regulation, and improve flight attendants’ SWB, so as to improve the overall service level of airlines and the market competitiveness of air transport.

This study has the following research limitations: (1) As the samples were all obtained from one airline, due to restrictions on the number of airline employees, research funding, and the nature of flight attendant work, the sample size collected in this study is relatively small, and the sample composition is mainly female. (2) Due to the particularity of the flight attendant working environment and the nature of their work, the results of this study may not be suitable for generalization to other groups, leading to the low universality value of the research results. (3) The analysis of mindfulness, spirituality, and SWB only stays at the level of the overall scale and does not analyze their sub-dimensions.

In future research, we will expand the sample collection range, increase the sample survey on flight attendants of other airlines, and conduct in-depth research on three factors of flight attendants’ mindfulness, spirituality and SWB, and further explore whether LKM has the same effect on samples of other groups to test the generalizability of the results of this study. In future studies, the mechanism of LKM’s promotion of spirituality and SWB will be further explored. Since the relationship among mindfulness, spirituality and SWB is relatively complicated, we need to conduct in-depth research on each factor and integrate various variables, so as to have an in-depth discussion on the relationship among mindfulness, spirituality and SWB. Interventions to improve people’s SWB are of interest to health policy makers because they may help reduce the prevalence of physical and mental disorders. However, from the perspective of public health, the training cost of face-to-face LKM is too high, and with the large number of mobile app users today, mobile apps have become a promising way for public health intervention. So LKM training can be done through distance learning. In the future, the development of a LKM app may be an effective, easily accessible and cost-effective way to reduce costs.

## 5. Conclusions

Our study revealed that LKM elicited a significant increase in spirituality and SWB. This result clarified the psychological effects of LKM and suggested a possibility of clinical use. The mechanisms which underlie the effects of the LKM on mindfulness, spirituality, SWB, and other psychological constructs require further elucidation.

## Figures and Tables

**Table 1:** Demographic characteristics of participants.

Characteristic	Total	LKM Group	Control Group
Age (SD)	29.26 (6.12)	30.14 (5.77)	28.37(6.39)
Male (%)	22 (22.4%)	9 (18.4%)	13 (26.5%)
Female (%)	76 (77.6%)	40 (81.6%)	36 (73.5%)

No demographic characteristic was significantly different among the two groups.

**Table 2:** Means and standard deviations for each measure of each group, pre and post assessment.

Group	Measures	Mean (SD)		
		Pre	Post	Post-Pre
LKM	SAIL	3.776 (0.421)	4.015 (0.475)	0.239 (0.490)
	SMS	3.787 (0.475)	3.856 (0.634)	0.069 (0.735)
	SWB	4.136 (1.491)	5.383 (1.474)	1.247 (1.518)
Control	SAIL	3.847 (0.472)	3.822 (0.432)	−0.025 (0.480)
	SMS	3.750 (0.485)	3.790 (0.436)	0.040 (0.460)
	SWB	4.089 (1.508)	4.255 (1.608)	0.166(1.306)

SMS, State Mindfulness Scale; SAIL, Spiritual Attitude and Involvement List; SWB, Subjective Well Being.
